# Thyroid hormone deprivation creates an immunological signature in the mouse liver, involving Kupffer cell presentation as the mouse ages

**DOI:** 10.1007/s12020-026-04619-2

**Published:** 2026-05-02

**Authors:** Helena Kerp, Devon Siemes, Janine Golchert, Georg Homuth, Uwe Völker, Lars Christian Moeller, Daniel Robert Engel, Dagmar Führer, Denise Zwanziger

**Affiliations:** 1 Department of Endocrinology, Diabetes and Metabolism, University Hospital Essen, University Duisburg-Essen, Essen, Germany; 2 Department of Immunodynamics, Institute for Experimental Immunology and Imaging, University Duisburg-Essen, Essen, Germany; 3 Interfaculty Institute for Genetics and Functional Genomics, University Medicine Greifswald, Greifswald, Germany

**Keywords:** Animal model, Age-Related Pathology, Endocrinology, Hormones

## Abstract

**Purpose:**

Aging is associated with an increased prevalence of chronic liver diseases suggesting impaired immune and metabolic function. In addition, thyroid hormone (TH) impacts liver physiology and TH deprivation or excess negatively affect organ maintenance. However, whether age-dependent consequences of TH alterations are reflected in a liver-specific adaptation is unknown so far. The present study aimed to characterize the impact of TH deprivation or excess on the liver transcriptome during aging.

**Methods:**

Five- and 21-month-old male C57BL/6 mice were exposed either to chronic TH deprivation or to chronic TH excess and compared to control treatment by microarray-based liver transcriptome analysis.

**Results:**

Significant roles of both TH state and age became obvious: Bioinformatic analysis of the liver transcriptome data revealed an age-dependent immune signature by chronic TH deprivation, an age-dependent immune and metabolic signature independent of exogenous TH modulation, as well as an age-dependent metabolic signature by chronic TH excess. Published data of single cell transcriptomic atlas characterizing aging tissues in the mouse were compared with our data and revealed Kupffer cell presentation in the immunological signature by TH deprivation during aging. Literature data for four prominent differentially expressed genes, namely *C1qb*, *C3ar1*, *Ctss*, and *Msr1*, revealed that the complement system, extracellular matrix remodelling, as well as the proinflammatory phenotype of Kupffer cells are altered by TH deprivation during aging.

**Conclusion:**

In conclusion, our study illuminates the interplay between TH deprivation, aging, and liver transcriptome signatures, highlighting potential implications for immune function and tissue maintenance, particularly through the modulation of Kupffer cell presentation.

## Introduction

The aging process of an organism is accompanied with an increased prevalence of chronic liver diseases [1–4]. Several hallmarks of aging including e.g. genomic instability, loss of proteostasis, mitochondrial dysfunction and cellular senescence impact liver homeostasis and can lead to impairment of liver function [5]. These hallmarks are thereby affecting all types of liver cells, parenchymal (hepatocytes) and non-parenchymal (e.g. Kupffer) cells. Moreover, in the aged liver metabolic- and immune-related changes were reported. For parenchymal cells, previous studies showed that the number and volume of hepatocytes decreased by aging which is associated with a lower intracellular NAD^+^ concentration impacting e.g., triglyceride and lipid metabolism [6]. For Kupffer cells, a higher cell number in aged as compared to young livers and an increased phagocytic activity as well as lower levels of autophagy were observed [6]. These data provide evidence that cellular composition and intracellular signaling of the liver are altered during aging.

Thyroid hormone (TH) signaling and homeostasis influence the aging process due to its multivariable function [7]. THs regulate organ development, growth, maintenance, as well as cellular metabolism [8]. Thyroid dysfunctions of hypo- and hyperthyroidism, associated with altered TH serum concentrations, negatively affect liver function and metabolism [9, 10]. In addition, the prevalence of thyroid dysfunction increases in older people, which increases the likelihood of liver damage [11–13]. The TH status of the aged livers in experimental mouse models was shown to be in a TH deficient-like state compared to the livers of young animals [14, 15]. Furthermore, TH deprivation contributes to the development of liver diseases such as metabolic dysfunction-associated steatotic liver disease (MASLD) and metabolic dysfunction-associated steatohepatitis (MASH) [16, 17]. However, it is still unknown whether age-dependent consequences of TH alterations are reflected in a liver-specific adaptation.

To characterize the potential impact of age on alterations in mouse liver transcriptome caused by TH deprivation or excess, we performed an experimental setup which imitates the human situation of “natural aging” with the occurrence of thyroid dysfunction. In different life-stages of male C57BL/6 mice (5 month and 21 month) chronic TH deprivation or excess were induced by exogenous manipulation. Bioinformatic analysis of microarray-based liver transcriptome data disclosed an age-dependent immune and metabolic signature by chronic TH alterations. Integration of our data into published single cell transcriptomic atlas from the Tabula Muris consortium [18] revealed Kupffer cell presentation in the immunological signature by TH deprivation during aging.

## Materials and methods

### Animals and study design

Male C57BL/6NTac mice (Taconic Europe A/S, Denmark) aged five and 21 months were housed in temperature- (23 ± 1 °C) and light-controlled (inverse 12:12 h light-dark cycle) conditions. Food and water were provided *ad libitum*. All animal experiments were performed in accordance with the German regulations for Laboratory Animal Science (GVSOLAS), the European Health Law of the Federation of Laboratory Animal Science Associations (FELASA) and Directive 2010/63/EU. The protocols for animal studies were approved by the *Landesamt für Natur*,* Umwelt und Verbraucherschutz Nordrhein-Westfalen* (LANUV-NRW).

Chronic TH deprivation and TH excess were induced as previously described [19, 20]. Briefly, for induction of chronic TH deprivation, animals were fed a low-iodine diet (TD.95007, Harlan Laboratories, USA) and received drinking water supplemented with 0.02% methimazole (301507, Sigma-Aldrich, USA), 0.5% sodium perchlorate (240931, Sigma-Aldrich, USA) and 0.3% saccharine as sweetener (240931, Sigma-Aldrich, USA). For induction of TH excess, i.p. injections of 1 µg/g body weight thyroxine (T2376, Sigma-Aldrich, USA) were performed every 48 h. In addition, animals under TH deprivation and control animals (without exogenous TH modulation) received i.p injections of solvent control (phosphate buffered saline) every 48 h. Animals under TH excess and control animals were fed a control diet (TD.95007 with added potassium iodide of 0.0012 g/kg: TD.97350).

### Liver collection

Animals were deeply anesthetized by i.p. injection with 10 µl/g body weight of a mixture containing 600 µl 10% ketamine and 200 µl 2% xylazine (Ceva, Germany). For liver collection, mice were perfused with heparinized saline through a needle placed in the left ventricle 24 h after the last thyroxine treatment or continuous TH deprivation as previously described [20, 21]. Livers of mice were isolated, shock frozen in liquid nitrogen and stored at -80 °C until further processing.

Preparation of hepatic RNA and microarray-based liver transcriptome analysis.

Hepatic RNA extraction (*n* = 4 independent tissue samples per experimental group), RNA quality control, further processing of RNA samples, including hybridization with GeneChip Mouse Gene 1.0 ST Arrays (Thermo Fisher Scientific Inc., Waltham, USA), as well as raw data extraction were performed as previously described by Lietzow et al. [22]. Shortly, total RNA was extracted from frozen liver tissues using TRIzol reagent combined with homogenization in a bead mill dismembrator, followed by further column purification and quality control.

### Data analysis, statistics, and visualization

Transcriptome raw data (gene-specific signal intensities) was normalized using quantile normalization and subsequently analyzed for differential regulation by two-tailed t-test, log_2_ fold change (log_2_FC) and signal-to-noise ratio (SNR). Log_2_FC was computed as $\:{\mathrm{log}}_{2}\left(\frac{mea{n}_{1}}{mea{n}_{2}}\right)$ and SNR with $\:\frac{mea{n}_{1}-{\mathrm{m}\mathrm{e}\mathrm{a}\mathrm{n}}_{2}}{{\mathrm{s}\mathrm{t}\mathrm{d}}_{1}+{\mathrm{s}\mathrm{t}\mathrm{d}}_{2}}$. Resulting effect sizes were visualized using heatmap with clustering, volcano plot and principal component analysis (PCA) employing the Python packages matplotlib (v.3.8.1) [23], seaborn (v.0.13.2) [24] and scikit-learn (v.1.3.2) [25]. The hierarchical row clustering for the heatmap was computed with the farthest neighbor-clustering algorithm from scipy (v.1.11.3) [26] on the pairwise distance matrix. Individual clusters were determined using a threshold of 0.65 times the maximum of all distances. Enrichment analysis for each cluster was performed using terms from the mouse specific Molecular Signatures Database (MSigDB) category M5-GO “Gene Ontology gene sets” (m5.go.v2023.1) and clusterprofiler (v4.6.2) [27–29]. For each cluster, the most enriched theme from biological processes was annotated next to the heatmap. Gene set enrichment analysis (GSEA) was generated by sorting genes according to SNR and using clusterprofilers GSEA function with the same terms used for enrichment analysis. String analysis was done with preselected genes, p-value < 0.05 and absolute log_2_FC > 1 for TH deprivation and control condition and not significant for TH excess, and the StringDB protein-protein interaction analysis graph together with the metrics of the most enriched terms from gene ontology biological processes were extracted [30]. Genes in the protein-protein interaction graph related to the immune system and macrophages according to enriched gene sets were highlighted in blue and red, respectively. The published Tabula Muris Senis scRNAseq dataset [18] was used to elucidate specific liver cell populations related to immune and macrophage signatures by using scanpy (v.1.9.8) to import the author supplied h5ad file (“59d1e3e3-834c-4f13-a002-dcfe084a04d6.h5ad”) and plot the intensities of target genes on the precomputed umap [31].

Literature research using PubMed of the four genes *C1qb*, *C3ar1*, *Ctss*, and *Msr1* revealing the following terms was performed: “gene+Kupffer cell”, “gene+macrophages”, “gene+liver”, “gene+aging”, “gene+knock-out mouse model”, and “gene+thyroid hormone”.

## Results

### Age and TH status affect the liver transcriptome

**Fig. 1:**
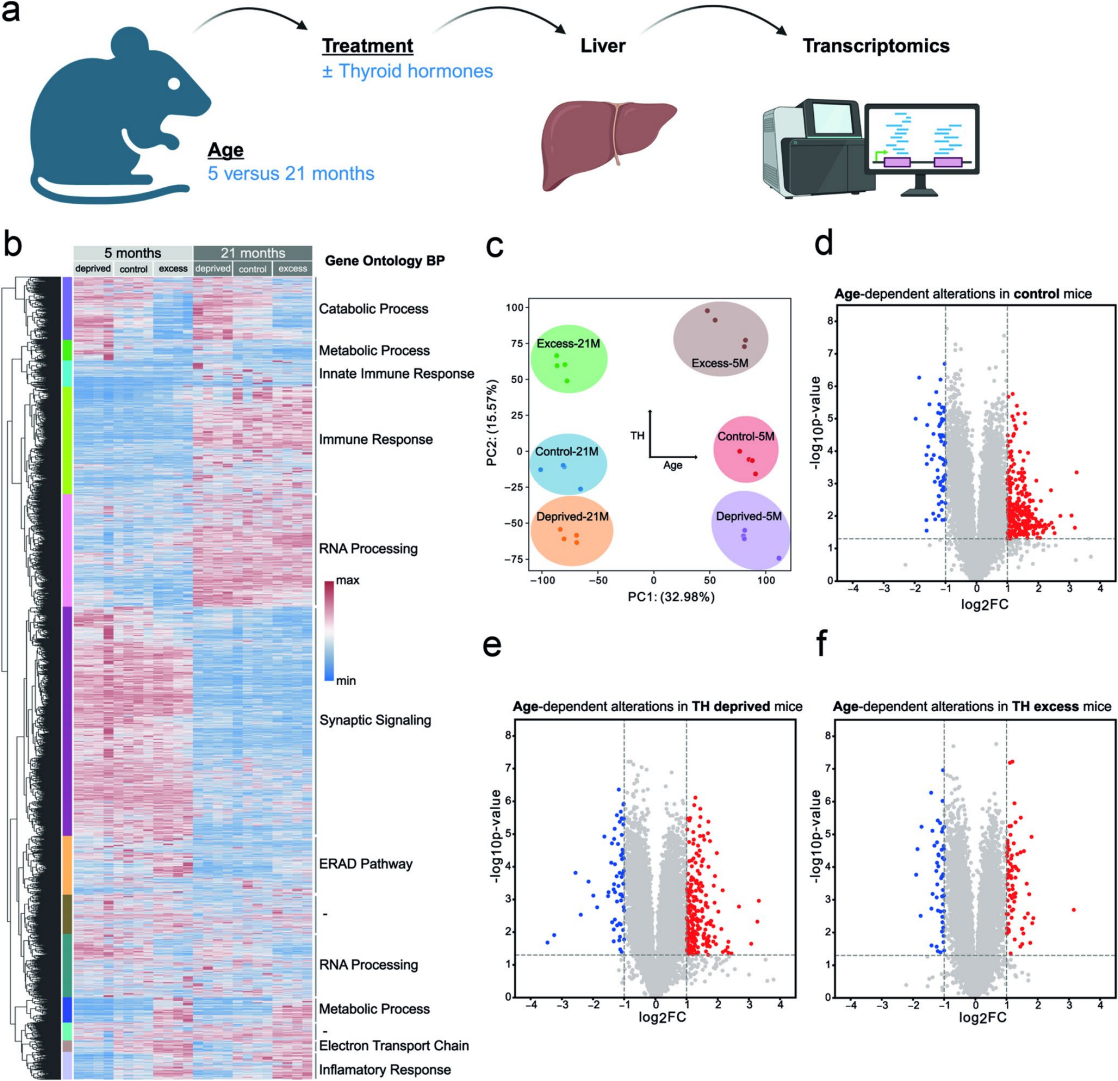
Age and thyroid hormone status affect hepatic transcriptome signatures. (**a**) Liver tissue of 5- and 21-month-old male C57BL/6 mice was analyzed by microarray-based transcriptome analysis. (**b**) Heat map with hierarchical clustering was generated with seaborn clustermap using the farthest neighbour clustering algorithm on the computed pairwise distance matrix. Clusters were determined using a threshold of 0.65 times the maximum of all distances. Enrichment analysis was performed with each cluster. The most enriched Gene Ontology terms (Biological process) are annotated. (**c**) Dimensionality reduction through principal component analysis (PCA) using all genes as input. (**d-f**) Volcano plot displaying the regulation of genes indicated by -log_10_ q-value and log_2_FC. Genes with q < 0.01 were colored blue and red to indicate significant down- and up-regulation, respectively, in (**d**) control condition, (**e**) thyroid hormone (TH) deprivation and (**f**) TH excess of 5- versus 21-month-old mice

To investigate age- and TH-dependent alterations in the hepatic transcriptome landscape, we compared the hepatic transcriptomes of 5- and 21-month-old mice after chronic TH deprivation and TH excess with corresponding control animals (Fig. 1A). Heatmap analysis of all derived gene-specific mRNA levels with hierarchical clustering indicated age- and TH-dependent gene expression clusters, which were associated with an enrichment of the following functional categories: “catabolic and metabolic processes”, “innate immune response”, “immune response”, “RNA processing”, “synaptic signalling”, “ERAD pathway”, “RNA and metabolic processing”, “electron transport chain”, and “inflammatory response” (Fig. 1B). PCA using all genes as input revealed age as first component (PC1), and thyroid dysfunction as second component (PC2) (Fig. 1C). The visualization of the p-values and log_2_FC among the 5- and 21-month-old mice under control condition (Fig. 1D), TH deprivation (Fig. 1E) and TH excess (Fig. 1F) indicated significantly less abundant (blue dots) and more abundant (red dots) transcripts (p-value < 0.05, absolute log_2_FC > 1) in the older animals. Notably, a stronger age-dependent differential gene expression, especially more abundant transcripts, was observed under TH deprivation as well as control condition as compared to TH excess. Thus, the data revealed that both age and TH affect transcript levels in the murine liver, with distinct expression patterns that are dominated by age.

### Age-dependent transcriptomic alterations indicate TH-conditioned immune and metabolic signatures

**Fig. 2:**
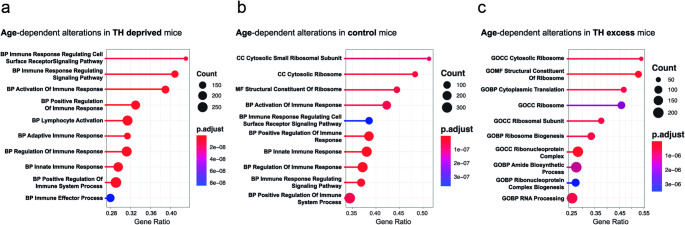
The thyroid hormone status shapes immune and metabolic signatures in the liver. Liver tissue of 5- and 21-month-old male C57BL/6 mice was analyzed by transcriptomics. Age-dependent Gene Set Enrichment Analysis was performed using the signal-to-noise ratio. (**a-c**) Age-dependent alterations are shown in (**a**) thyroid hormone (TH) deprivation, (**b**) control condition and (**c**) TH excess

To further analyse the differences between the gene signatures hallmarking control condition, TH deprivation, and TH excess during aging, GSEA was performed for the hepatic transcriptome data sets of 5- versus 21-month-old mice (Fig. 2). GSEA revealed a progression in the gene signatures from TH deprivation over control condition to TH excess. In particular, age-dependent alterations under TH deprivation were solely related to the immune system with e.g. differences in the category “immune response” (Fig. 2A). Under control conditions, metabolic and immune signatures were affected by age, e.g. by differences in the categories “immune response” and “protein biosynthesis” (Fig. 2B), whereas under TH excess only metabolic signatures like the categories “protein biosynthesis” and “RNA processing” were affected by age (Fig. 2C). The integrative analysis indicated a progression in gene signatures from TH deprivation to TH excess, with pronounced alterations related to the immune system and general metabolism.

### Aging imprints the immunological signatures in Kupffer cells under TH deprivation

**Fig. 3:**
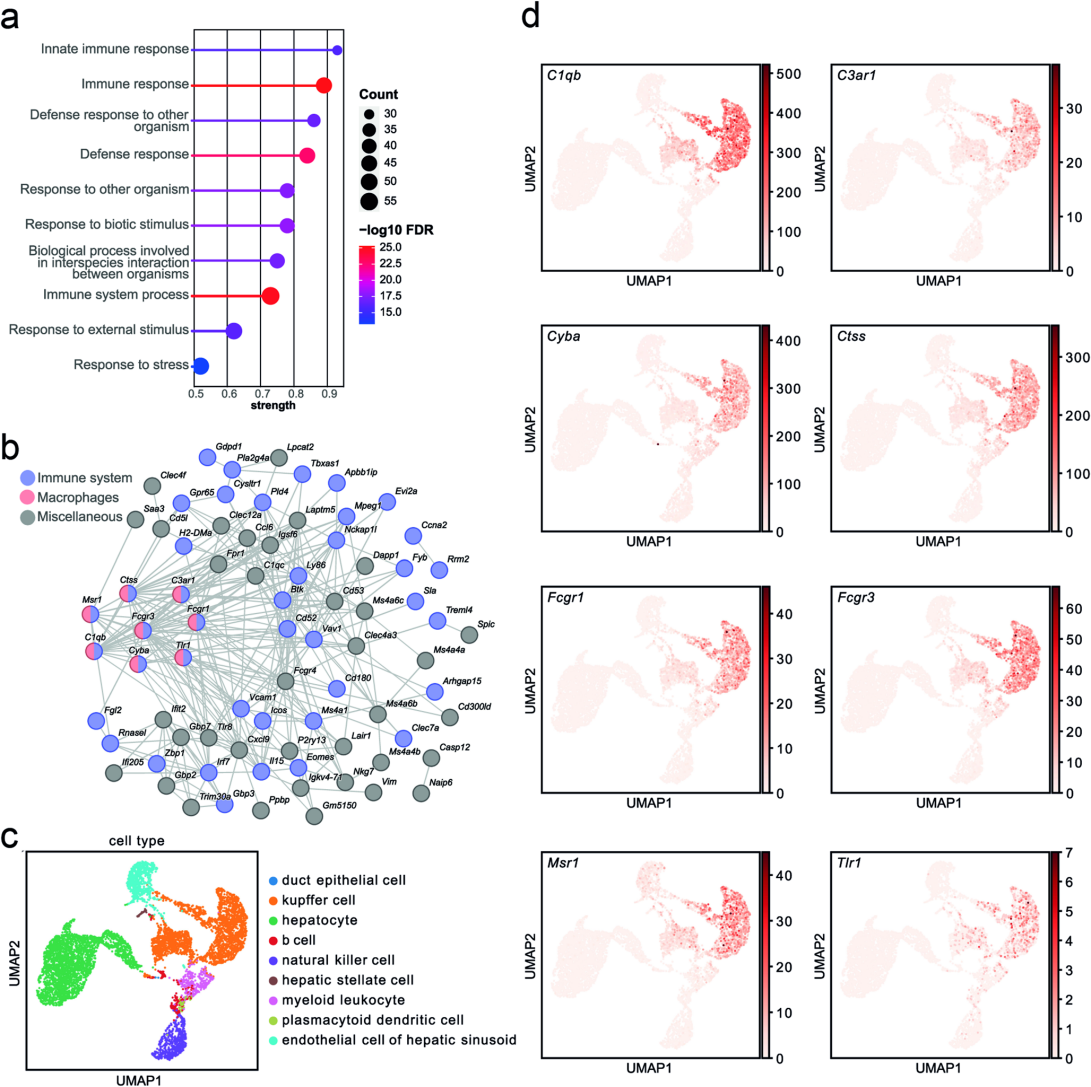
Liver transcriptome analysis indicates a Kupffer cell signature in thyroid hormone deprivation. Integrative analysis of the transcriptome data. (**a**) Top 10 Gene Ontology terms according to FDR from STRING enrichment analysis of all genes significantly upregulated (p-value < 0.05 and log_2_FC > 1) under thyroid hormone (TH) deficient and control condition but not under TH excess during aging of 5- versus 21-month-old male C57BL/6 mice. (**b**) STRING cluster of all genes increased in TH deficient and control condition during aging, but not by TH excess. Genes related to the immune system and macrophages are highlighted in blue and red, respectively. Edges connecting nodes represent protein-protein interactions according StringDB. (**c**) Cell type annotation of all liver cells from the Tabula Muris Senis scRNAseq atlas [18]. (**d**) Expression pattern of macrophage-related genes in the scRNAseq dataset shown in (c)

In order to analyse the age-dependent alterations of the immune signatures under TH deprivation and control conditions in more detail, the top 10 Gene Ontology terms from a STRING enrichment analysis of significantly upregulated genes under TH deficient and control but not under TH excess during aging were analysed and revealed critical immune response processes (Fig. 3A). STRING cluster analysis of all genes increased in TH deficient and control condition but not under TH excess during aging, identified eight genes (*C1qb*, *C3ar1*, *Cyba*, *Ctss*, *Fcgr1*, *Fcgr3*, *Msr1*, and *Tlr1*) associated with the categories “immune system” and “macrophages” (Fig. 3B). To elucidate specific hepatic cell populations related to immune and macrophage signatures under TH deprivation and control conditions, cell type annotation of all liver cells from the Tabula Muris Senis scRNAseq atlas [18] was used (Fig. 3C). The expression pattern of the eight genes related to the immune system and macrophages revealed a Kupffer cell signature (Fig. 3D).

## Discussion

In the global human population, the proportion of elderly individuals is increasing and the impairment of liver function with aging exacerbates the risk to develop chronic liver diseases and increases disease severity. During aging per se, the liver undergoes a gradual decrease in volume and blood flow associated with an increased fat and cholesterol content and an impaired regenerative capacity [6]. Moreover, TH deprivation contributes to the development of liver diseases such as MASLD and MASH [16, 17]. In the present study, we performed unbiased transcriptome profiling of the aging liver with and without TH modulation to obtain insights into age-dependent consequences of altered TH levels. Our analysis revealed an age-dependent immune signature by chronic TH deprivation, an immune and metabolic signature without exogenous TH modulation and a metabolic signature by chronic TH excess. Moreover, in the immunological signature by TH deprivation during aging Kupffer cell mediated antigen presentation was observed by comparison of our bioinformatic data to the Tabula Muris Senis scRNAseq dataset [18].

The liver is composed of many immune cells including macrophages. These macrophages are divided into distinct populations, liver resident macrophages (Kupffer cells) and monocyte-derived macrophages recruited from the blood [32]. Although the comparison of our bioinformatic data to the Tabula Muris Senis scRNAseq dataset revealed Kupffer cell presentation involvement of the proteins encoded by the above-described genes, we cannot exclude that also other cell types like non-resident macrophages contribute to the gene expression. However, recent data suggest that most macrophages in liver physiology are Kupffer cells and that these cells not only contribute to immune surveillance but also to lipid metabolism of the liver [6, 32]. Moreover, in liver pathology Kupffer cells are considered to be proinflammatory and contribute to disease development, whereas these cells are gradually lost during disease progression [32]. Interestingly, many studies investigated the topic of aging and macrophages, but there are only limited data on the effect of aging in Kupffer cells. It is described that Kupffer cell number and their phagocytic capacity increases during aging [6]. Thus, the question arises if the more pronounced immunological signature under TH deprivation as well as control conditions during aging is due to increased expression of the corresponding genes or an increase of the hepatic Kupffer cell population. This spatiotemporal aspect should be investigated in further studies.

Our data identified TH-associated immune signatures in the mouse liver during aging with eight key candidate genes (*C1qb*, *C3ar1*, *Cyba*, *Ctss*, *Fcgr1*, *Fcgr3*, *Msr1*, and *Tlr1*) showing more abundant transcripts under TH deficient condition during aging. For four of these genes’ literature research was performed allowing discussion of gene expression profiles in the context of aging and TH deprivation. The proteins encoded by *C1qb* and *C3ar1*, namely complement complex 1 subunit q chain b and complement 3a receptor, both represent components of the complement system. During aging, C1q abundance is increased which is possibly related to the Wnt/β-catenin signaling pathway and may reduce tissue regeneration of aged cells [33]. Furthermore, increased C1q abundance might indicate liver injury as shown previously during liver fibrosis in cirrhotic rats or liver diseases in humans [34]. *C3ar1* encodes a G-protein coupled receptor, which participates in multiple signaling pathways, e.g. Wnt/β-catenin and p38 MAPK signaling [35]. *C3ar1* expression was thought to be restricted to the innate immune system but the receptor can be also found in other cell types like endothelial cells [36]. Moreover, *C3ar1* knockout mice exhibit a transient resistance to diet-induced insulin resistance and liver steatosis [37], a genetic observation confirmed by pharmacological intervention [38]. The complement system of tissue resident macrophages is involved in clearance of pathogens, immune complexes, and apoptotic cells from the circulation [39]. Kupffer cells lining sinusoids are exposed to a constant blood flow from the portal vein, which might sensitize these cells to systemic TH deprivation. Interestingly, in previous studies alterations of TH levels affected complement components in vitro and in vivo [40, 41]. The increased hepatic transcript levels of *C1q* and *C3ar1* in our study might indicate an altered complement system during aging and its potential involvement in tissue maintenance of the liver.

Cathepsins are responsible for e.g. extracellular matrix (ECM) remodeling, autophagy and immune signaling. As lysosomal enzymes, cathepsins control homeostatic and pathological processes. Cathepsin S (CTSS) physiology differs from the physiology of other cathepsins due to the association of CTSS to antigen-presenting cells with macrophages as a major cell source [42]. Interestingly, CTSS levels are increased in the fibrotic livers of patients and rodent models [43]. Moreover, it was shown that macrophage-derived CTSS remodels the ECM to promote liver fibrogenesis [43]. This is in line with the finding that liver fibrosis is caused by excessive accumulation of ECM proteins due to chronic inflammation. *Ctss* knockout mice are protected from carbon tetrachloride-induced liver fibrogenesis associated with a reduced hepatic infiltration of macrophages and expression of inflammatory cytokines [43]. These data support not only the implication of CTSS as marker for diagnosis of liver fibrogenesis or as therapeutic approach by pharmaceutical intervention but also as target of TH deprivation during aging.

The macrophage scavenger receptor 1 encoded by *Msr1* is highly pleiotropic and involved in several physiological and pathological processes throughout diverse tissues [44]. MSR1 is important in innate and adaptive immune regulation. In the liver, it is enriched in Kupffer cells and triggers the uptake of lipids leading to the formation of foamy Kupffer cells with a proinflammatory phenotype. The *Msr1* transcript levels are significantly associated with the incidence of hepatic steatosis, cirrhosis, and hepatocellular carcinoma in patients with MAFLD and increases with disease progression [45]. In high-fat diet supplemented mice, *Msr1* deficiency attenuated the lipid-induced inflammatory response in the liver, suggesting that lipid accumulation in tissue-resident macrophages by MSR1 is a trigger to recruit immune cells [45]. These data indicate expression of *Msr1* as an immunological imprinting of Kupffer cells and critical sensor for lipid homeostasis during aging and TH deprivation. Age-independently, it could be speculated that MSR1 expression is reduced by TH deprivation due to diminished TH signaling in macrophages. In general, there is a positive correlation between MSR1 expression and liver diseases [45]. In the TH deficient scenario, a reduced lipid export combined with a diminished beta-oxidation could accelerate lipid accumulation thereby promoting liver diseases like MASLD.

It is well known, that aging and TH alteration impair liver physiology and contribute to the development of chronic liver diseases. In conclusion, the present study points towards a critical interplay of metabolic and immunologic processes in TH deprivation in the aging liver.

## Data Availability

The data that support the findings of this study are available from the corresponding author upon reasonable request.
